# Exploring the Impact of Internet Use on Memory and Attention Processes

**DOI:** 10.3390/ijerph17249481

**Published:** 2020-12-17

**Authors:** Josh A. Firth, John Torous, Joseph Firth

**Affiliations:** 1 Department of Zoology, Edward Grey Institute, University of Oxford, Oxford OX2 8QJ, UK; joshua.firth@zoo.ox.ac.uk; 2 Merton College, University of Oxford, Oxford OX2 8QJ, UK; 3 Department of Psychiatry, Beth Israel Deaconess Medical Center, Harvard Medical School, Boston, MA 02215, USA; jtorous@bidmc.harvard.edu; 4 Division of Psychology and Mental Health, University of Manchester, Manchester M13 9PL, UK; 5 NICM Health Research Institute, Western Sydney University, Westmead, NSW 2145, Australia

**Keywords:** cognitive processes, digital health, digital technology, mHealth, neuroscience, social media

## Abstract

The rapid uptake of the internet has provided a new platform for people to engage with almost all aspects of life. As such, it is currently crucial to investigate the relationship between the internet and cognition across contexts and the underlying neurobiological mechanisms driving this. We describe the current understanding of this relationship across the literature and outline the state of knowledge surrounding the potential neurobiological drivers. Through focusing on two key areas of the nascent but growing literature, first the individual- and population-level implications for attention processes and second the neurobiological drivers underpinning internet usage and memory, we describe the implications of the internet for cognition, assess the potential mechanisms linking brain structure to cognition, and elucidate how these influence behaviour. Finally, we identify areas that now require investigation, including (i) the importance of the variation in individual levels of internet usage, (ii) potential individual behavioural implications and emerging population-level effects, and the (iii) interplay between age and the internet–brain relationships across the stages of development.

## 1. Understanding Internet–Brain Relations

In recent years, the internet has become an integral aspect of everyday life for most adults and adolescents [1,2], producing a global shift in how people search for and share information, connect with one another, obtain social recognition and rewards, and acknowledge social status. This has become particularly pronounced with the rise of smartphone technologies, which offer constant internet access and encourage individuals to remain always connected to the online world.

Along with the vast range of possibilities of harnessing the internet for improving the self and society, the potential risks of extensive internet usage are also becoming evident [3]. Specifically, various lines of research have now begun to examine if, and how, this widespread and extensive internet usage may adversely impact our cognitive processes and how changes in brain functioning may underpin these effects [4]. However, the majority of studies to date have only investigated specific online activities in isolation, making it difficult to draw overall conclusions. Nevertheless, some attempts have recently been made to synthesize the broad literature surrounding this area [4], and a framework of primary areas of cognitive functions (in relation to the internet) has been established as including (i) social cognition, (ii) attentional processes, and (iii) memory storage/retrieval.

While modern synthesis and reviews have summarised the previously available evidence for the impact of the internet on cognition [4], further examination is still required to update and draw further attention to more recent, emerging aspects of how internet usage can impact cognitive processes. Indeed, while the relationship between the internet and social cognition is without a doubt an important area (see Box 1), the processes of attention and memory are currently experiencing rapid growth in terms of developing our understanding of how cognition is affected by internet usage. Such growth is particularly driven through research into these two cognitive processes providing new insights into the specific implications of internet-related cognitive changes at the individual and population level and the neurobiological mechanisms that underpin these apparent relationships.

Box 1 **Social cognition.**

Although not the primary focus of this review, the interplay between the internet and social cognition is referred to across various contexts in this synthesis, as this area of research is a particularly rapidly developing topic across fields and is allowing various powerful empirical studies due to the vast number of data available within this area, particularly from harnessing social media. The current framework characterizes the overlap between online social networks and real-world sociality as a “new playing field for the same game” [4]. Although social-networking platforms on the internet clearly provide unparalleled potential for social interactions, a substantial body of literature demonstrates that core socio-cognitive processes and social network structures integral to “real-world sociality” are reflected in online social networks [5,6,7,8,9,10]. The strong correlations between neural processes implicated in both online and offline social interactions further illustrate how “artificial” aspects of social media platforms (e.g., the quantitative metrics of popularity, such as likes of posted content and explicit friendship requests/acceptance) can have “real-world” social consequences [4,11]. For instance, compelling evidence has recently demonstrated that being subjected to online rejection evokes the same neural responses as those seen in “real-world’ rejection [11,12,13,14] Thus, by presenting an artificial environment that so closely interacts with regular sociality, online social networks have the potential to interfere with a broad range of cognitive processes involved in social comparisons, self-evaluations, and even mental health [11,15,16,17,18,19]. Attempts to now understand how the relationship between internet usage and social cognition may carry-over into affecting other cognitive processes (such as attention and memory as referred to across this review) will be of great use for increasing knowledge on the cross-contextual implications of the internet.

First, for attention, the perpetual flow of information provided by the internet may potentially interfere with sustained concentration, by prompting people towards “media multi-tasking” between different types of incoming sources of information [4]. Indeed, the available data support the hypothesis that engaging in excessive media multi-tasking reduces performance in sustained concentration tests [20,21,22]. Additionally, recent experimental studies have found that even brief interaction with hyperlinked websites can produce notable immediate reductions in concentration capacities; deficits that can persist for a short duration even after ceasing internet usage [23].

Secondly, memory is another cognitive process that may be impacted by the internet, due to the persistent access to factual information afforded by ubiquitous internet access. The internet may act as a “superstimulus for transactive memory” [24] by tilting us towards an over-reliance on the online world as an endless, and always available, source of external memory storage. Supporting this, a number of empirical studies have found that using the internet for information-gathering tasks does accelerate the process but appears to fail in recruiting certain patterns of brain activation important for long-term storage of the retrieved information [25,26].

In this paper, we aim to further examine the mechanisms through which internet usage may influence human cognition, particularly with regards to focusing on the recent findings around the impact of internet usage on attention and memory, due to the rapid and constant growth of research in these two areas. To do this, we build on the evidence presented in previous reviews [4] to firstly utilise the evidence and literature surrounding the internet affecting attentional capacities, primarily through describing how extensive internet usage and the mass of information afforded from this may predispose us towards media-multitasking and divided attention, at the level of both the individual and the population/society. Secondly, we use the recent findings from the emerging literature on memory processes to examine the neurobiological mechanisms that may underlie internet-induced alterations in memory, with particular emphasis on the important indication that these relationships may vary across different age groups. Third and finally, we highlight the promising topics within these areas and how further investigation of these will benefit the wider field. Reviewing all evidence, we offer recommendations for how the potentially adverse effects of internet usage could be ameliorated or avoided using emerging evidence.

## 2. The Impact of Internet Use on Attention

The relationship between the internet and attention processes is experiencing relatively rapid development; as such, it is particularly important that regular synthetic updates are considered. Although there is clearly a broad array of processes through which the internet could impact core cognitive processes (i.e., as outlined above), its influence on attention has recently provided an especially rich knowledge base through which the digitalised world may have cognition-related implications for the individual and also potential population-level effects on attention changes. Interestingly, at both of these levels (i.e., individual and population), there is an emerging, but largely independent, demonstration that the actual extent of internet usage (rather than just access to it) is an important factor for shaping cognition. In this context, we discuss the influence of internet usage for cognitive processes in the individual and then consider how this also relates to population-level implications. In doing so, we hope to emphasise that, while it may be particularly challenging to assess how individual-level implications of internet usage may scale directly to shape societal level outcomes, it is becoming apparent that attention processes may provide an in-road into investigating these phenomena.

### 2.1. Individual-Level Implications

**Figure 1:**
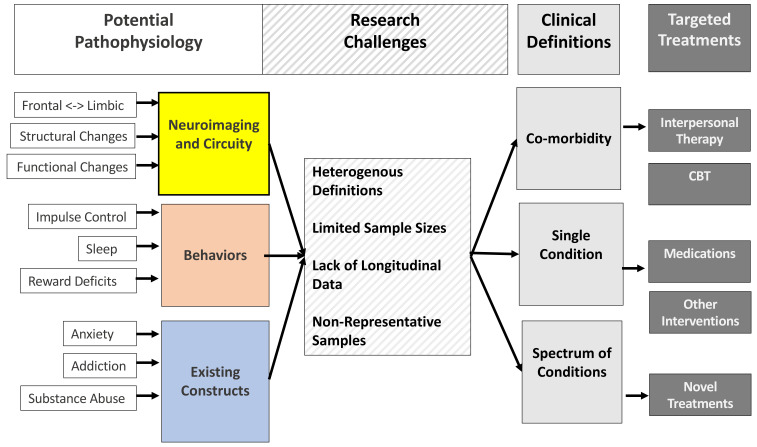
Synthesis of potential underpinning processes, risk factors, and treatments of internet usage disorders, along with contemporary challenges faced by current research within this area. The Targeted Treatments “CBT” box also includes internet-delivered cognitive behavioural therapy (termed iCBT) [28].

While it is straightforward to conceptualise “internet usage” as a dichotomous variable or separate individuals into users vs. non-users, the intricacies of internet usage differ hugely between individuals. First, the most strikingly variable that would be expected to moderate the effects of internet usage on the brain would be the quantity of use. Indeed, a large sum of research has investigated the correlates of the highest levels of internet usage and dependence, specifically in those with “internet use disorders” (IUDs). Indeed, a growing amount of research is beginning to advance the understanding of the processes underpinning IUDs, the risk factors, and the potential treatments (as synthesised in Figure 1), and, although not the primary topic of this review, this area now provides a specifically useful and applied avenue for elucidating the consequences of the quantity of internet usage on individual-level attention. For instance, a 2017 review [27] identified structural changes or deficits in brain regions associated with attentional control, reward processing and motivation in those with IUDs compared to healthy controls. IUD-related deficits in these brain regions associated with cognitive control also appear to be reflected, or even manifest, in the heightened rates of related behavioural deficits associated with IUD. It would be particularly interesting to examine how quantity interacts with quality/type of usage (e.g., social media usage vs. online gambling usage) and its impact on IUD.

With regards to effects on attention/concentration, a vast sum of research has shown strong links between excessive usage of the internet and IUDs (i.e., Figure 1), and this is now beginning to be linked to attention-deficit hyperactivity disorders. Most notably, a 2017 systematic review examined the link between IUDs and ADHD across 15 independent studies (2 cohort studies and 13 cross-sectional studies) [29] and found that individuals with IUDs had over a 3 times higher likelihood of ADHD than healthy controls. Even after adjusting for potentially confounding factors, the adjusted odds ratio for ADHD in IUD individuals remained clinically and statistically significant (OR = 2.51, 95% C.I. = 2.1 to 3.0). In further examinations of the data, the meta-analysis also found that IUDs are also associated with more severe symptoms of ADHD, separately from the diagnosis of ADHD as a clinical condition. For example, “inattention” scores were much higher in those with IUDs compared to control samples (standardised mean difference of 0.84, 95% C.I. = 0.65–1.02). Subsequently to the 2017 review, a more recent cross-sectional study confirmed the association between ADHD and IUDs in a sample of 1000 university students and professional online gamers [30].

Despite this strong observational link, the question of course remains whether excessive use of the internet is a causal factor for ADHD, or whether adolescents with probable ADHD are more susceptible to excessive internet usage. Future research, particularly research using methods that allow investigation of the causality and direction of relationships, will provide much-needed insights into the potential adverse impacts of the extensive internet usage on attentional disorders in young people [31,32]. However, due to the saturation of the internet across the globe, it is difficult to examine the causal relations between extensive internet usage. Nonetheless, a recent study by Loh et al. [33] capitalised upon a rare sample of 35 young adults in India with minimal prior contact with Internet-related technologies to experimentally investigate the impact of one month of unlimited Internet access on neurocognition. Results showed that introducing high levels of internet usage to this previously naïve sample increased media-multitasking behaviours in just one month, significantly more than a comparison group of internet-familiar young adults measured over the same timeframe. Therefore, the individual-level attention effects of the internet are clearly providing a body of evidence showing that consideration of the quantity of usage is vital for understanding these relationships as well as having applied implications in terms of psychological syndromes (here ADHD). Continued research within this area, as well as further studies examining the context and quality of usage, is now necessary to advance general understanding of these concepts.

### 2.2. Emerging Population-Level Effects

Modifications to individual-level behaviour (often through cognitive changes) can ultimately carry over to shape the emergent population-level processes that arise as a product of the actions of the individuals within the society. As such, it is perhaps intuitive (but nevertheless of much importance) that the possible effects of the internet on divided attention appear to extend beyond considering individuals that are vulnerable to developing specific attentional disorders and appear to also apply on a population scale. However, combining a fine-scale assessment of internet-related cognitive changes at the individual-level with the large-scale examination of the consequences of such changes for the population is particularly challenging, which means that, currently, such “holistic” empirical studies are limited. Nevertheless, one of the most compelling pieces of evidence for this is provided by a recent study examining the effects of the internet on “collective attention span”. referring to the amount of attention a popular topic receives on a population level [34]. Across various types of online content, the study found strong evidence that, over time, shorter intervals of collective attention are given to individual topics. For instance, the study first examined 24 h usage of the top 50 most-used Twitter hashtags across the world (sampled across 43 billion tweets), and how this changed over time. Results showed that whereas a highly popular hashtag stayed within the top 50 for 17.5 h in 2013, this gradually decreased over time, with top 50 hashtags maintaining their position for only 11.9 h by 2016. These patterns persisted across a range of online and offline topics of public interest and across different timeframes [34].

On a further note, this study [34] provides a clear demonstration that the internet is not only providing pathways for modifying population-level cognition but also enables the almost-real-time quantification of such phenomena. Therefore, although it is often problematic to infer causative effects from population-level observations, such large-scale and detailed data enables inferences of population-level effects that would otherwise be difficult to quantitively identify. Indeed, this study also employs a simple mathematical model to illustrate a basic mechanistic process of how increased production and subsequent consumption of information cause population-level attention span (and topic turnover rates) to shorten. Importantly, this mechanistic model explains the observed data well, suggesting that the ever-faster flows of collective attention were primarily driven by the increasing flow of information across the internet (i.e., total rates of internet-based informational content production and consumption) and that the abundance of information available today is indeed shortening the attention spans of the population. While further studies to determine the population-level effects of internet usage are needed, a fully detailed account of how these population-level consequences arise also requires further understanding of individual-level effects. Thus, empirical studies that monitor individual cognitive changes in response to individuals’ internet usage, while simultaneously measuring population-level outcomes, will provide new insights into how the effects of sustained internet usage on an individual level can manifest in changes in human cognition on a population-scale.

## 3. Neurobiological Underpinnings of Internet–Brain Relations

An emerging body of cross-sectional research indicates that certain aspects of internet usage and online behaviours may be responsible for changes in brain structure and neural functioning, independent of “offline” versions of such behaviours. For instance, in the context of social cognition (Box 1), neuroimaging studies have shown that individuals’ number of Facebook social connections (their “Facebook friends”) can predict the grey matter volume of particular brain regions (such as in the right entorhinal cortex [6]), while their real-world social networks (real-world friends) holds little relationship with grey matter volume in these regions. Similarly, large amounts of internet usage [35] and particularly media-multitasking [36] are correlated with reduced grey matter volume in the anterior cingulate cortex and other prefrontal regions associated with sustaining concentration/ignoring distractor stimuli [35,36]. However, these cross-sectional studies fail to determine if these neurophysiological alterations are a cause or consequence of different types of internet usage and do not allow causal inferences. Indeed, it is currently almost impossible to establish the long-term neural changes induced by engaging with the internet on a regular and sustained basis due to the relatively recent adoption of the internet as the prime source of information consumption in our society. Nonetheless, despite these challenges, memory processes are appearing to be proving to be a particularly fruitful line of research, due to the vast amounts of knowledge surrounding the fundamental neurological underpinnings of memory, as well as the ability to empirically test this process within individuals [25].

### 3.1. Internet Use and Memory Processes

Given the sheer amount of time the average individual spends engaging in online activities (of both communicative and non-communicative nature), various insights into neurobiological pathways through which online activities affect cognition can be gained from investigating the shorter-term effects of internet usage on the brain [3,36,37,38,39]. Existing neuroimaging studies examining the acute or short-term effects of internet-based information processing/consumption currently report mixed results. On one hand, an emergent body of literature indicates that the unprecedented potential for finding additional (or alternative) information at the push of a button could interfere with the retention of the information sought. To examine the neurobiological mechanisms through which this may occur, experimental studies have compared internet searching to encyclopedia-based information retrieval. These studies have found that internet searching can lead to reduced activation in brain regions associated with working memory [26,40] and alterations in functional connectivity of memory retrieval circuits [41]. Furthermore, large quantities of internet use are associated with a reduced volume of the brain regions associated with cognitive control, hypothetically due to the internet usage encouraging high levels of flicking between information sources (i.e., media multitasking) at the expense of brain circuitry used in sustained concentration [35,36].

Separately to media multitasking, another pathway through which online information sources may interfere with regular memory processes is linked to the constant accessibility of this external mass of information, which could potentially train a reliance towards informational retrieval over informational retention [42]. Some studies have recently reported that internet search training may increase behavioural impulses towards internet use by impacting upon brain regions involved in reward, attention, and inhibitory control [43].

Furthermore, since the internet essentially acts as an external or transactive memory system, this allows for “cognitive off-loading” of certain cognitively demanding tasks, such as semantic memory retrieval [25,44]. This, in turn, may free up cognitive resources, which can instead then be reallocated towards the use and development of higher-level cognitive abilities [45]. In line with this, internet search training has recently been shown to facilitate neural connectivity by increasing white matter integrity [46].

### 3.2. Age Interactions in Internet–Brain Relations

Alongside examining the biological mechanisms that potentially govern the effect of the internet on the brain, there is an increasing indication that consideration must be given to how the effects of internet usage may differ across age groups. For instance, cross-sectional studies in older adults have shown that those who engage in more internet/email activity have greater performance in memory recall [47]. This positive effect is possibly due to the cognitive stimulation from the wealth of information provided by internet usage, facilitating the retention of cognitive capacities in ageing. As such, research reporting that the reward, attention, and inhibitory control regions of the brain are negatively influenced by internet search training (which holds consequences of internet-usage behavioural impulses) may not be entirely generalizable, as these studies did not involve older individuals [43]. Neuroimaging studies conducted in older adults who use the internet regularly show that internet-based information retrieval uses greater amounts of neural circuitry than text-based information, specifically in regions implicated in multiple higher-level cognitive functions, including decision making and complex reasoning [48]. However, there is a paucity of observational or experimental studies examining how long-term internet use could produce sustained changes in brain development, connectivity, and structure, and how this may underlie internet-induced alternations in typical cognitive processes. Of note, there is a particular dearth of research in children and adolescents, whose younger brains may be more responsive to the potential neuroplastic (i.e., flexible neurological dynamics) changes associated with increased internet use. To our knowledge, only one existing prospective study has examined the associations between internet usage and brain development in youth. Specifically, the study compared brain development over a three-year time period in children with frequent internet use vs. children with low/no internet use [49]. The results suggest impeded development of verbal intelligence in the young people who engaged in the highest levels of internet use over the three years. Furthermore, the neuroimaging aspect of the study indicated a feasible neurobiological mechanism through which this may occur, as higher frequency of internet use was linked to a reduction in the ageing-related increases in both gray and white matter volume, brain regions which are linked with the development of executive functions, language, and attentional control [49].

## 4. “What Now?” Research Priorities for Future Investigations

The latest synthesis (Parts 2 and 3 above) of the current research examining how the internet may influence cognitive processes (particularly attention and memory) also provides insight into the topics that currently hold unrecognized potential for furthering our understanding of how cognitive processes are shaped by internet usage. Here, we outline how prioritizing further work in the areas of establishing the long-term implications for cognitive processes, and understanding the context of internet use. We conclude by looking beyond the primary risks of internet usage for cognition towards discussing the positive implications the internet can hold for cognitive functioning.

### 4.1. Examining Long-Term Impacts of Internet Use

Currently, the findings of studies examining the acute effects of internet usage are mixed, perhaps due to the wide variety of different online behaviours studied to date. Within the existing literature, there is increasing evidence to indicate that using the internet extensively for factual information retrieval and media-multitasking may impact adversely on brain regions associated with memory (i.e., through long-term storage) and attention (i.e., through sustained concentration). However, the long-term effects of the internet on brain structure, and how this underpins downstream effects on cognitive capacities, remain unknown. In particular, future studies aiming to elucidate the neurobiological pathways through which internet usage impacts cognition, including memory and attention, must also consider the potential interaction of these effects with age. Specifically, it should be considered that whereas internet usage may facilitate cognitive stimulation in older people, it could also adversely impact the development of higher-level cognitive capacities in youth. Alongside this, further consideration must be afforded to how various types of internet usage (i.e., different online behaviours and styles of using the internet) may ultimately determine the outcomes of internet usage for individuals.

### 4.2. Putting Cognitive Effects of Internet Use in Context

While much research is being pursued in understanding how the quantity of internet usage may influence cognitive processes, a less-frequently addressed question is, “what types of internet usage affect cognition, and in what ways?”

As outlined above, the two primary contexts of internet usage are information consumption and social communication/interactions. Importantly, differentiating these two outlets may be crucial for furthering our understanding, particularly as social usage of the internet is known to have a range of distinct effects. For instance, a major draw to the internet for some is to engage in virtual communities in order to exchange information, social support, and friendship [50]. A further draw is the ability to be able to express thoughts and feelings to large audiences via these communities, social networks, online groups, or bulletin board systems. As such, the relevance, and the strength, of the relationship between online social activity and that of the real world (see Box 1), along with the potential consequences of this, is widely recognized as a topic of great interest [51,52]. There is also some indication that online social interaction influences human cognition in ways analogous to real-world socialization (e.g., see Box 1) and that brain regions linked to social cognition and associative memory are also correlated with online social network size [6]. Indeed, memory capacity may act as a key determinant of online social networks due to the large number of potential associates an individual may hold [6]. Further, cross-sectional studies have shown excessive social media use to be associated with decreased grey matter volume in regions related to emotional regulation and social cognition, including the bilateral amygdala and right ventral striatu [53]. High daily Facebook use has also been linked with reduced nucleus accumbens grey matter volume, a structure associated with motivation, reward, learning, and addiction [54].

Clearly, commercial online social media platforms such as Facebook, Instagram, and Snapchat have a remarkable capacity to engage users [32,55], and the exceptional aspects of internet-based social networks bring into question whether the distinctive properties of the online world will hold negative or positive ramifications for users’ well-being. As it stands, it is currently unknown if and when engaging in online social networks is overall of benefit or risk to general mental health. On the one hand, increasing the potential for social interaction and expression appears to be beneficial. On the other hand, extensive usage may divert time away from “real-world” social interaction time (see Box 1) and other beneficial lifestyle behaviours such as physical activity and sleep [56]. Further examination of the costs and benefits of internet usage in this context is now needed. To ameliorate any adverse effects of internet usage on brain functioning and mental health, there is a need for more fine-grained research to be conducted using real-time monitoring to establish what types/amounts of internet usage may be detrimental to well-being, along with determining if and how potential adverse may be mediated through other factors (e.g., through excessive internet usage being tied to victimization, social withdrawal, or excessive sedentary behaviour). Following the acquisition of such informative data, national and international health organizations could formulate evidence-based guidelines on types and amounts of internet usage (for different age groups) in a similar way to public health guidelines for other health behaviours, e.g., physical activity and sleep [57,58]. These could then be disseminated across the population such that the public can make informed decisions regarding engaging in this relatively new facet of our lifestyles in an informed and safe manner.

Alongside this, it is important to gain further understanding of how social and non-social (e.g., information-gathering) processes influence cognitive processing differently. However, it should be considered that these contexts are often non-independent in reality. This non-independence is generated by any activity that links these contexts within individuals. For instance, individuals will often socially share information that they initially accessed in a non-social context. Furthermore, links between the social- and non-social aspects of the internet could be created through information gained in a non-social context, subsequently shaping an individual’s social interactions (in terms of how they interact, and how often they engage in interactions) or through social outlets (such as social media sites) being used as sources of searching for information (instead of non-social searching platforms). Therefore, exploring the non-independence and the interactions between these contexts, and the consequences for cognition, is now of great interest.

### 4.3. Discovering Avenues for Beneficial Effects of Connected Technologies

The evidence reviewed here has largely concerned the unintentional psychological consequences of internet use. Along with the ongoing efforts to understand the impact of the internet on human thought processing and social behaviours, there is also a rapidly growing body of work examining how we can capitalize upon this in a positive way. The most longstanding example of this is internet-delivered cognitive behavioural therapy (iCBT), which is delivered remotely via a computerized interface and has been shown to reduce psychological symptoms of various disorders with similar efficacy to face-to-face therapy [28]. Although completion and ongoing adherence to these initial internet interventions efforts have proven challenging in real-world settings, the dawn of smartphone technologies presents a novel platform for constantly accessible, easily disseminated and user-friendly internet-based psychological interventions [59]. Evidence from meta-analyses has already demonstrated some efficacy of smartphone-delivered therapies for reducing both depression and anxiety [60]. However, the extent to which benefits observed in these trials are due to active components of the therapies themselves, as opposed to individuals connection with their smart devices and expectations for benefit, has yet to be fully determined [61].

## 5. Summary and Conclusions

In conclusion, the introduction of the internet has clearly impacted many diverse aspects of society. We hope this review further contextualises the current findings linking the internet to the brain, cognition, and behavioural outcomes, while also highlighting the key areas for further research in an era of rapid digitalisation. Whereas the effects of internet use on the brain are not yet fully understood, there is convergent evidence from multiple fields that our extensive interactions with this novel feature of society could influence our attention, memory, and other aspects of cognition. Further longitudinal work is required, particularly in young people. Nonetheless, as we continue to refine our understanding of potential adverse consequences of internet usage, now is also the time for examining how this revolutionary tool can be utilized to produce improvements in psychological and cognitive health.
